# Supplementary material for: Chromothripsis during telomere crisis is independent of NHEJ, and consistent with a replicative origin
Source: Genome Res. 2019 May;29(5):737–49. doi: 10.1101/gr.240705.118 (PMC6499312; doi:10.1101/gr.240705.118)
Supplement: Supplemental Material [file supp_gr.240705.118_Supplemental_file_1.zip › contigs/annotated_contigs/DB50/contig.2.DB50_length_276_mean_cov_4.34782608696.docx]

**DB50_length_276_mean_cov_4.34782608696**

```
AGTTAAGAGTGAGAACATGCAGTGTTTGGTTTTCTGTTCTTGTGTTAGTTTGCTGAAAATGATGGTTTCCAGC|AAAATTAT|ATTTTA
 >chr18:35848445-35848518 - E=1e-26                                               >chr1
TCTAAAATATAATTATTTCAACATATAACCAATATAAAATTATGAATGAAATATTACATATTTTTCATACTAAGTCTTTGAAATCCATT
0:27193331-27193526 - E=2e-106
GTGCATTGTATAGTTCAGGTACATCTCAGCTTGGACTAACCACATATCAAGTGTTCAATAGCTACACATAGGTAAGTGGCTACTGGTTT

GAACAGAAACG
```
